# Development of a machine learning-based survival prediction model for ALS inclusive of the advanced-stage population

**DOI:** 10.1080/21678421.2026.2652322

**Published:** 2026-04-13

**Authors:** DANIELLE BEAULIEU, KELLY SMITH, CAMPBELL ROSS, SYLVIA YIP, TANIA C. FELIZARDO, CHRISTINA FOURNIER, JONATHAN D. GLASS, JAMES D. BERRY, DANIEL H. FOWLER, DAVID L. ENNIST, THE POOLED RESOURCE OPEN-ACCESS ALS CLINICAL TRIALS CONSORTIUM

**Affiliations:** 1 Origent Data Sciences, Washington, DC, USA; 2 Rapa Therapeutics, Rockville, MD, USA; 3 Emory University, Atlanta, GA, USA; 4 Mass General Hospital, Boston, MA, USA

**Keywords:** Amyotrophic lateral sclerosis, survival, respiratory insufficiency, gradient boosting machine model, vital capacity

## Abstract

**Objective:**

Develop a machine learning-based model for survival prediction in ALS, including advanced-stage patients (≤50% predicted normal vital capacity [VC_50_]).

**Methods:**

Training data from the PRO-ACT Database (*n* = 6896) was supplemented with advanced-stage ALS patients (*n* = 678), with model validation on distinct advanced-stage ALS patients (*n* = 403). Baseline patient characteristics, including slopes from symptom onset, were used to train a random forest model to identify parameters with the greatest relative importance (RI) for predicting survival outcomes. These parameters were used to train a gradient-boosting machine (GBM) model that generated patient-level survival predictions (log-hazard). Model discrimination and calibration were quantified by C-index and calibration-in-the-large plus calibration slope, respectively. Kaplan-Meier curves were generated, with patient stratification into tertiles based on the predicted survival risk score.

**Results:**

Baseline characteristics with the highest RI for driving survival predictions included: VC% slope (20.2%); age (12.4%); VC% (9.9%); VC(L) (7.5%); ALSFRS-R (6.6%); and ALSFRS-R slope (5.1%). Model performance upon external validation was satisfactory for both discrimination (C-index, 0.709 [95% CI, 0.671–0.746]) and calibration (calibration-in-the-large, 0.083 [95% CI, −0.073–0.232]; calibration slope, 0.992 [95% CI, 0.789–1.198]). At 8-months from baseline, the model successfully stratified patients by survival prognosis, with low-, average-, and high-risk population tertiles having observed median survival probabilities of 85, 69, and 43%, respectively.

**Conclusions:**

This model accurately predicts survival prognosis in ALS, including patients with severely impaired respiratory function. This new understanding of patient-specific factors that drive survival prognostication will be invaluable for reducing patient heterogeneity in clinical trials evaluating novel therapeutic modalities in early- and advanced-stage ALS.

## Introduction

Amyotrophic lateral sclerosis (ALS) is a primary neurodegenerative process with a secondary neuroinflammatory component that accelerates disease progression (1), with a median time from diagnosis to lethality within three years (2). Nonetheless, there exists a great deal of heterogeneity in the prognosis of people living with ALS (pwALS), which relates at least in part to a multitude of initiating neurodegenerative events (3) and variations in secondary immune phenotypes (4,5). This heterogeneity has contributed to the inability of a great number of clinical trial efforts to identify new therapeutic modalities (6); currently, the standard-of-care for the majority of ALS patients consists only of riluzole, which was FDA-approved in 1995 and only extends survival by a few months (7). An insufficient ability to predict the survival of ALS clinical trial participants is problematic in the setting of both single-arm studies (due to sub-optimal historical control cohorts) and randomized controlled trials (RCT; due to insufficient stratification and resultant unequal risk probabilities in treatment *vs.* placebo cohorts). Because ALS is a rare, orphan, and uniformly lethal disease that lacks effective therapeutic options, there exists a great public health need to accurately prognosticate ALS clinical trial participants to more effectively evaluate the large number of potential agents that show promise for disease amelioration. This need for improved prognostication ability in ALS is widely recognized globally, as evidenced in part by a 2021 review article (8) that identified nineteen externally validated studies in this arena.

Over the past decade, in an attempt to address the obstacle of ALS heterogeneity, numerous studies have demonstrated the promise of machinelearning models that can predict survival, overall disease progression (ALSFRS-R scores), and progression of respiratory insufficiency (reduction in pulmonary vital capacity). This era was initiated by the Prize4Life contest, held to launch the availability of the PRO-ACT database of longitudinal ALS patient medical records (9). In 2016, a study (10) led by Origent Data Sciences identified that a random forest (RF) model yielded superior results relative to previous methodologies in terms of predicting disease progression (ALSFRS-R scores). This model was trained on baseline patient information in the PRO-ACT Database (11) and validated on an external dataset from the Emory University ALS clinic (12). It is important to note that this model was trained on data from patients with relatively early-stage disease, as defined by the following mean values: time since diagnosis (234days); ALSFRS-R score (37.8); and forced vital capacity (3.4L; translates into *>*80% of predicted normal). In additional studies involving early-stage ALS patients, the Origent survival model was identified as superior to traditional methods as a tool for randomized trial stratification (13) and applicable to inform the design of single-arm clinical trials evaluating interventions with a potentially large disease ameliorating effect (14). In 2018, Westeneng et al (15) aggregated data from 14 European ALS centers and developed a model for predicting a composite survival endpoint. More recently, additional models have been introduced for predicting survival in pwALS, including a 2023 model that supplemented clinical features with brain magnetic resonance images (16) and a 2025 publication that used a PRO-ACT Database propensity-matched control cohort that identified an increased survival time in recipients of oral edaravone (17). Taken together, these initial studies indicate that survival prognostication models represents a potentially powerful tool with the capacity to transform the design and interpretation of ALS clinical trials.

In spite of these advances, there is currently a paucity of published data regarding machine-learning prognostication in patients with advanced-stage ALS. Research in this advanced-stage population is rapidly emerging due in large part to the congressionally-mandated ACT for ALS (18), which supports investigational treatment for pwALS who are not eligible for conventional clinical trials, resulting in an increased number of expanded access programs (EAPs). This effort to improve access to experimental therapies is a critical goal because ~60% of pwALS are excluded from trial participation due in part to parameters of advanced-stage ALS, including low ALSFRS-R scores or greatly reduced respiratory vital capacity (values ≥50% of predicted normal are nearly universally required for ALS trial entry) (19). Given this situation, it is important to develop prognostication models that are applicable to advanced-stage ALS, with an emphasis on survival prediction. While survival alone has significant limitations as an endpoint in early-stage ALS clinical trials due in large part to the necessity for an increased trial duration (20,21), it predictably will be less of a limitation in trials evaluating advanced-stage ALS. Understanding the degree to which this limitation is mitigated in advanced-stage ALS patients represents an important objective of this project. The ability to incorporate survival as an endpoint for clinical trials involving advanced-stage ALS patients is particularly important given the known limitation of using ALSFRS-R and vital capacity slope estimations as endpoints in this patient population (22,23). One specific aim of the current model development relates to a prospective phase 2 clinical trial of RAPA-501 therapy in advanced-stage ALS patients (NCT06169176); in this trial, one research objective is to compare the survival of RAPA-501 recipients relative to a virtual control cohort generated by the model described in this manuscript.

Toward this goal, we developed a machinelearning model that predicts survival in pwALS, including those with advanced-stage disease, operationally-defined as pwALS who have vital capacity (VC) values of ≤50% of predicted normal at baseline. Specifically, our objectives were to develop a model that: (1) characterizes the degree to which differential survival predictions exist within this advanced-stage population; and (2) identifies the key baseline clinical characteristics that drive the survival predictions.

## Methods

### PRO-ACT database enrichment with advanced-stage ALS patients

**Figure 1.**
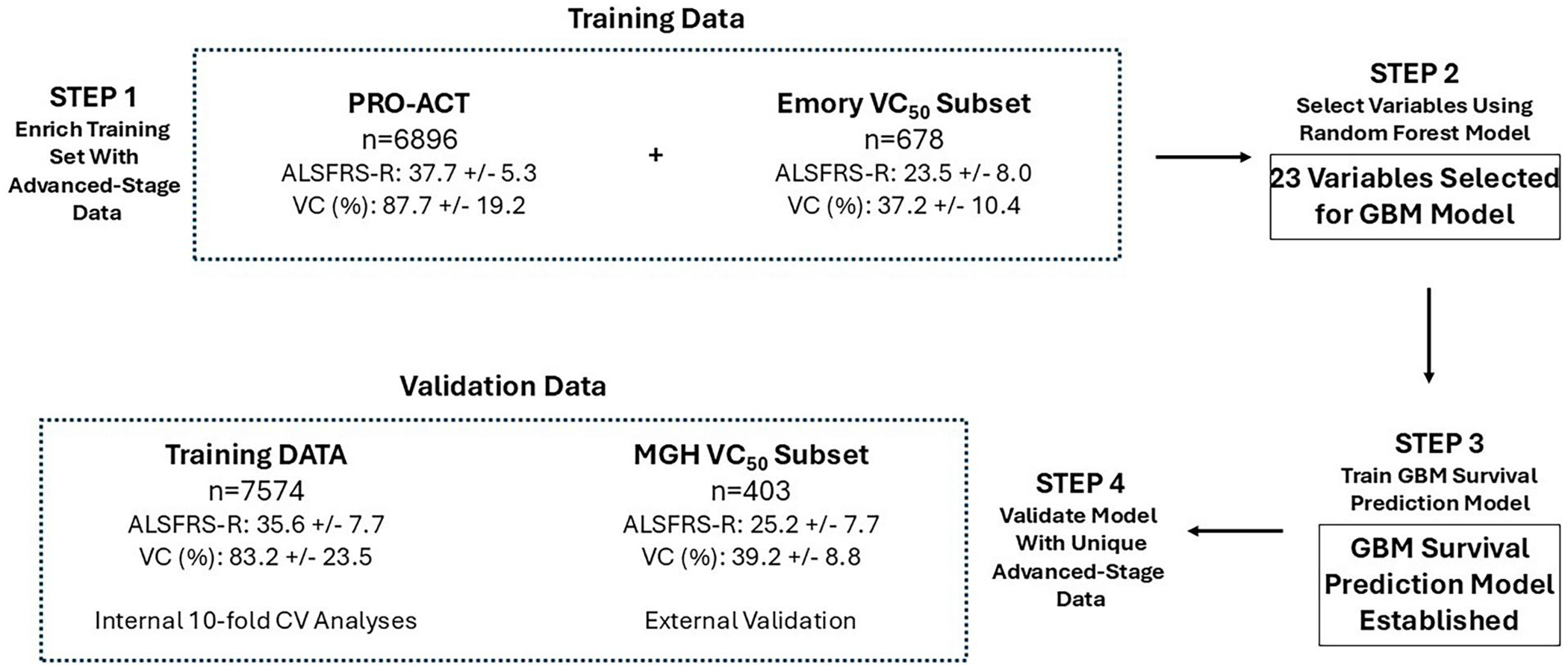
Development of an ALS survival prediction model inclusive of the advanced-stage population. In step 1, baseline characteristics on *n* = 6896 patients from the PRO-ACT Database (“PRO-ACT”) with relatively early-stage ALS were supplemented with 678 patients from the Emory University ALS clinic enriched for advanced-stage parameters (“Emory VC_50_ Subset”). Using this training dataset, a random forest model was used to select variables for the GBM model (step 2), with subsequent training of the GBM survival prediction model (step 3). Finally in the validation phase (Step 4), the model was tested internally using 10-fold cross validation on the training data and tested externally on an independent database of *n* = 403 patients enriched for advanced-stage parameters (“NHC VC_50_ Subset”). VC (%), indicates vital capacity as the percent of predicted normal value; values shown are mean ± SEM.

Model training and validation schemes are shown in Figure 1. Similar to an initial study (10), the PRO-ACT Database, which is comprised of data from placebo and control arms of completed clinical trials since 1990, was used as the backbone for model development. The dataset was restricted to the 6896 patients that met the inclusion criteria of ≤5 missing variables of the 23 included in the final model and the existence of post-baseline follow-up data. Because the PRO-ACT database is relatively deficient for patients with advanced-stage ALS, training data was supplemented with an additional 678 patients from the Emory University ALS clinic (12) that were treated from 2008 to 2023, met the aforementioned inclusion criteria, and also had ≤50% VC (“Emory VC_50_ Subset”). This resulted in a total training dataset of 7574 patients. The model was externally validated on a subset of patients (*n* = 403) from the ALS Natural History Consortium (ALS NHC) (24) that were accrued starting in 2015, met the inclusion criteria, and also had ≤50% VC (“NHC VC_50_ Subset”). Missing variables in the dataset were resolved through imputation (continuous variables, imputed with mean value of entire population; categorical variables, imputed with the highest frequency for that variable). Although multiple imputation methods that incorporate Rubin’s rules to aggregate estimates can produce optimal imputation (25), the methods we used are less computationally expensive and showed comparable performance in the current model relative to more complex imputation methods. For the Emory VC_50_ and NHC VC_50_ subsets, the first timepoint with a VC measurement of 50% or lower was taken as the baseline. Our use of data from the three databases was approved by the Institutional Review Board (IRB) of record at the relevant institution. Our research findings are being reported in accordance with updated TRIPOD guidance for transparent reporting of prediction models (26); please see the Supplementary Appendix for the TRIPOD-AI checklist.

### Inclusion of potential predictors for model development

Survival was measured from baseline to death; patients that did not die were censored at the last known time alive. Full available follow-up data was used; that is, there was no use of administrative censoring. Based upon availability in the PRO-ACT, Emory, and NHC databases, the following baseline variables were included as potential model parameters: vital capacity (VC), including both absolute VC (in liters, L) and percent expected VC (VC%), and slope of %VC decline from symptom onset to baseline; vital signs (pulse, blood pressure); demographic and morphologic features (age, height, weight, sex, and body mass index [BMI]); ALS disease history (days from symptom onset, days from diagnosis, site of onset, use of riluzole, gastrostomy); ALSFRS-R, absolute values (including total scores, grouped sub-scores, and answers to individual questions); and ALSFRS-R slope variables (as measured from symptom onset to baseline).

### Random Forest model and subsequent gradient-boosting machine (GBM) model

An internal data subset (randomly selected 80% of training data) was used to train a preliminary random forest (RF) model (25) to rank candidate variables as survival predictors using permutation-based relative importance (RI). The remaining 20% of the training data determined accuracy and precision to ensure acceptability of the RF model. Subsequently, the most important variables in the RF model were selected to train a gradientboosting machine (GBM) model to predict survival outcome (27). The GBM was trained using a semi-parametric Cox proportional hazards model with baseline hazard estimated non-parametrically from the observed event-time data. The model was validated internally with a 10-fold cross validation and then externally validated against the NHC VC_50_ Subset.

### Model assessment: discrimination and calibration

As previously delineated (28–30), C-index was used to assess the model’s ability to discriminate patients based on survival outcome. Calibration slope and calibration-in-the-large (CITL) were used to determine the fit between observed and predicted outcomes.

### Graphical assessment of model: forest plots; ROC curves; and Kaplan-Meier curves

As previously described (10), forest plots were used to display discrimination and calibration values and corresponding 95% confidence intervals. Receiver operating characteristic (ROC) curves (29,30) at timepoint *d* = 306 were used to show model discrimination, with area under the curve (AUC) calculated using the nearest neighbor approach. Day 306 was used because this value represents the elapsed study duration for the afore-mentioned phase 2 clinical trial of RAPA-501 therapy in advanced-stage ALS patients (NCT06169176). Kaplan-Meier (KM) curves (31) with 95% confidence intervals were generated, with separate curves for low-, average-, and high-risk tertiles stratified by predicted risk score (loghazards of event).

## Results

As shown in Table 1, the subset of the PRO-ACT database that met the inclusion criteria (*n* = 6896) was representative of ALS patients with relatively early-stage disease, as indicated by both mean ALSFRS-R scores (37.7±5.3 [SEM]) and vital capacity values (% of predicted normal, 87.7 ± 19.2). The Emory VC_50_ subset had advanced-stage parameters, with greatly reduced ALSFRS-R scores (23.5 ± 8.0) and vital capacity values (% VC, 37.2 ± 10.4). The external validation dataset (NHC VC_50_ Subset) was indeed representative of patients with advanced-stage ALS, with greatly reduced ALSFRS-R scores (25.2 ± 7.7) and vital capacity values (% VC, 39.2 ± 8.8). Notably, these values were significantly less than those of the combined PRO-ACT and Emory VC_50_ Subset training data (35.6 ± 7.7 and 83.2 ± 23.5, respectively). Please see Table 1 for additional information pertaining to these sets of data, including comparisons of: age; sex; time from onset; time from diagnosis; riluzole usage; limb *versus* bulbar onset; slope of decline of ALSFRS-R; gastrostomy procedure; deaths through day 306; and median survival from baseline, diagnosis, and symptom onset.

**Figure 2.**
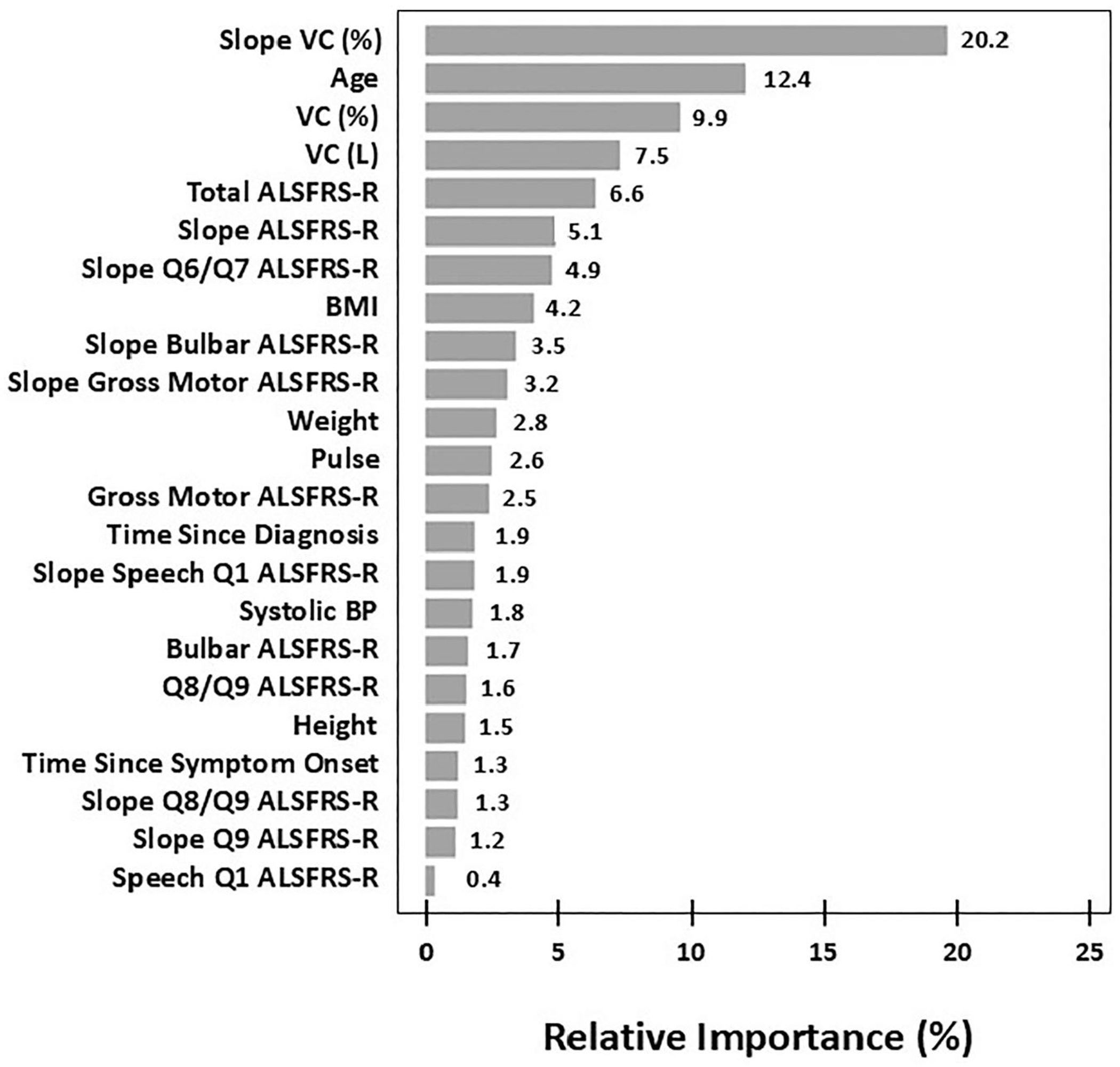
Relative importance (RI) of survival prediction for the 23 variables included in the final GBM model. These 23 variables were selected from the results of a preliminary random forest (RF) model trained on 80% of the training data that ranked the importance of potential predictors of survival. The remaining 20% of the training data was used to ensure that the RF model was performing in an acceptable range. Abbreviations: VC, vital capacity; L, liter; Q6/Q7, Q8/Q9, Q9, indicates importance of various subset(s) of the ALSFRS-R score; BMI, body mass index; BP, blood pressure.

Figure 2 shows that vital capacity measures ranked high in the relative importance (RI) for selected survival predictors derived from the results of the GBM model. Slope of %VC decline (20.2%) was by far the highest, while the baseline VC in both absolute and percent terms (7.5 and 9.9%, respectively) were 3rd and 4th highest. Age and total ALSFRS-R score were also in the top 5. The pace of ALS disease progression was a recurring driver of survival predictions, with 10 of the 23 identified parameters consisting of slope measurements (%VC; ALSFRS-R, both total and numerous subsets). Metabolic factors may have driven survival predictions, as evidenced by the inclusion of BMI (RI, 4.2%) and weight (RI, 2.8%).

**Figure 3.**
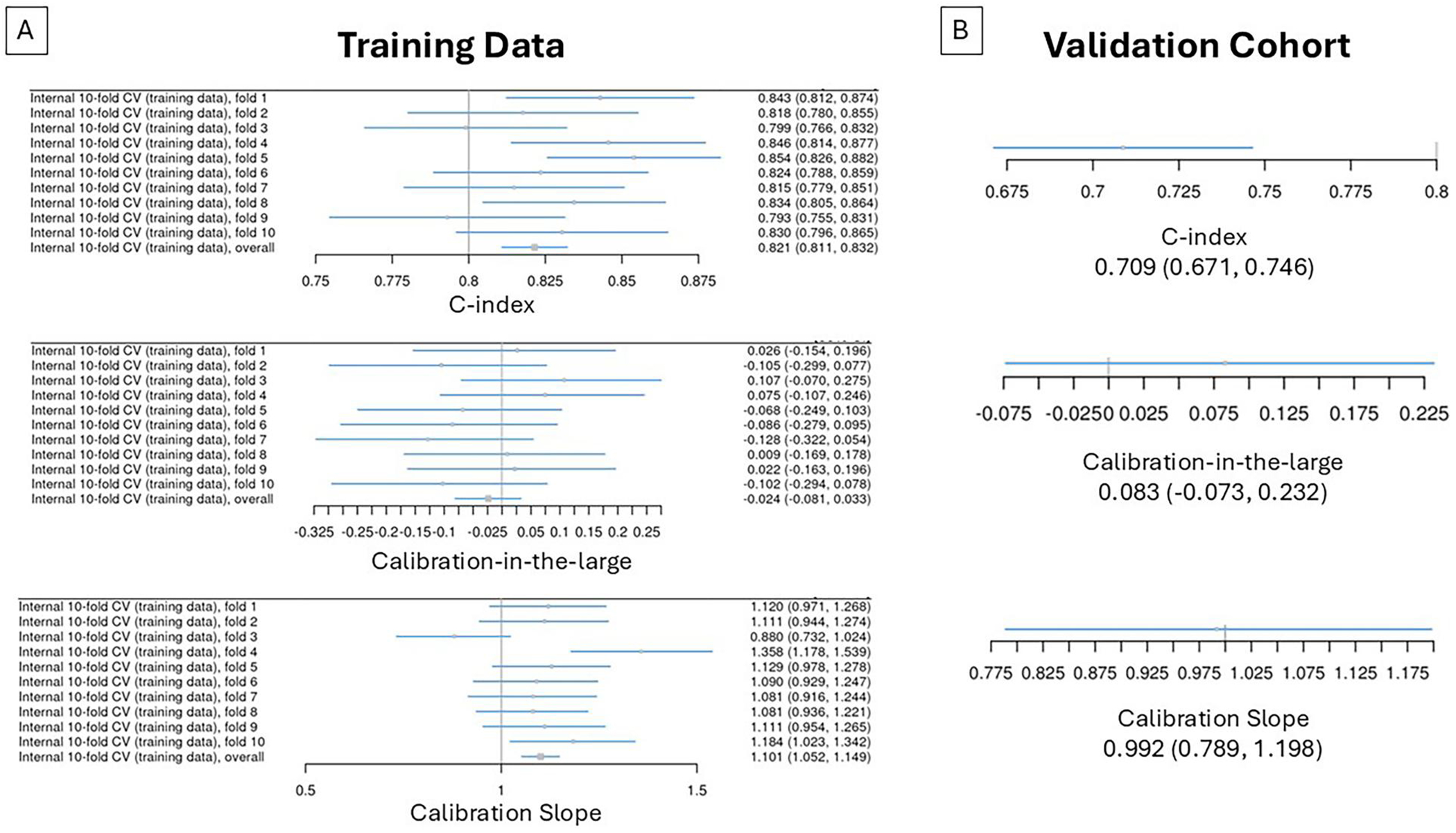
Model discrimination and calibration in the training data (A, internal 10-fold cross-validation [CV] studies) and external validation cohort (B). As illustrated in (A), 10 internal folds of the training data were evaluated for discrimination (C-index) and calibration (calibration-in-the-large intercept and calibration slope). (B) Shows the discrimination and calibration data for the validation cohort. Estimates and their 95% confidence intervals are shown.

**Figure 4.**
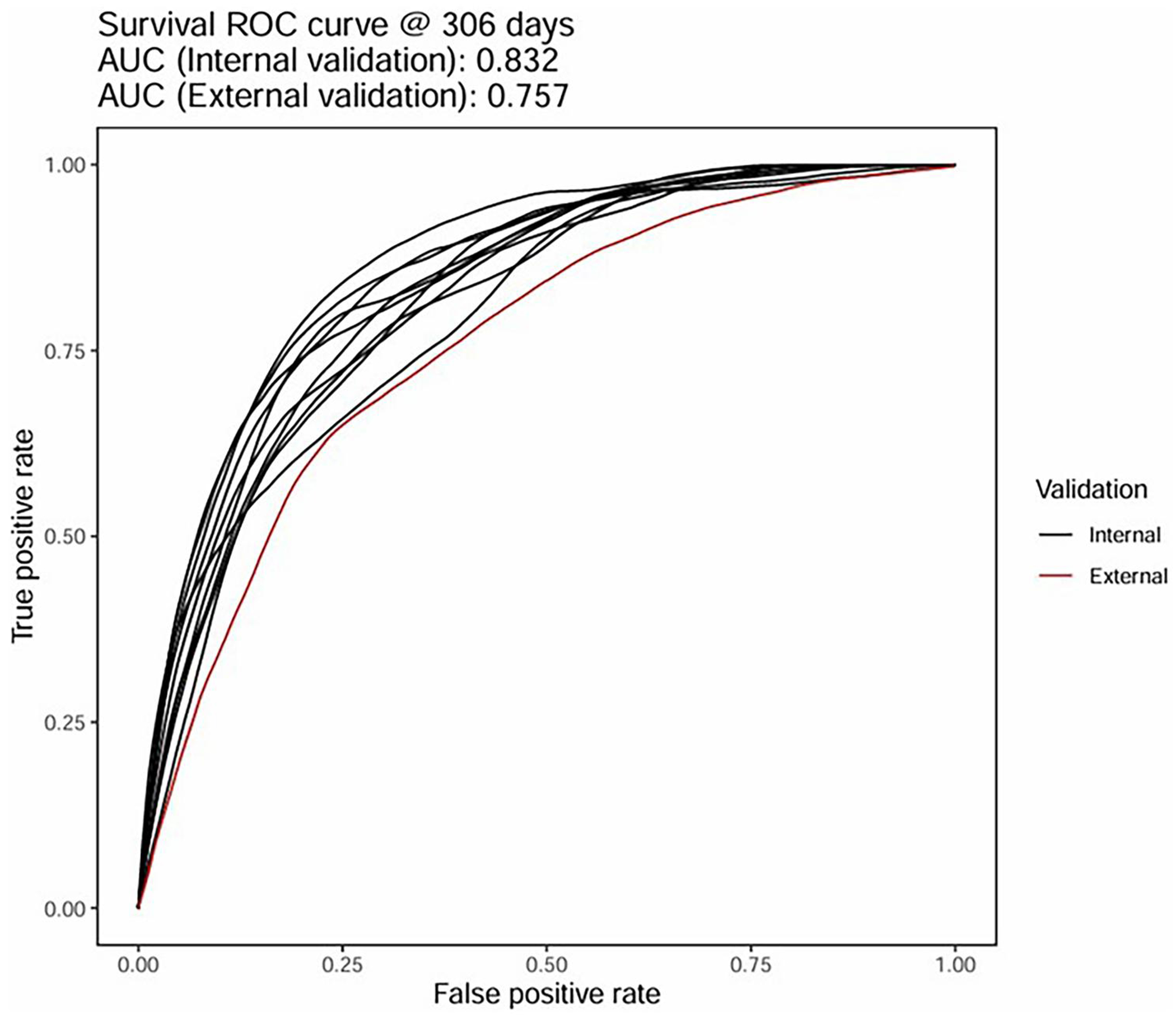
Receiver operating characteristic (ROC) curves for training data (black lines; one for each fold) and the validation cohort (red line) at day 306.

**Figure 5.**
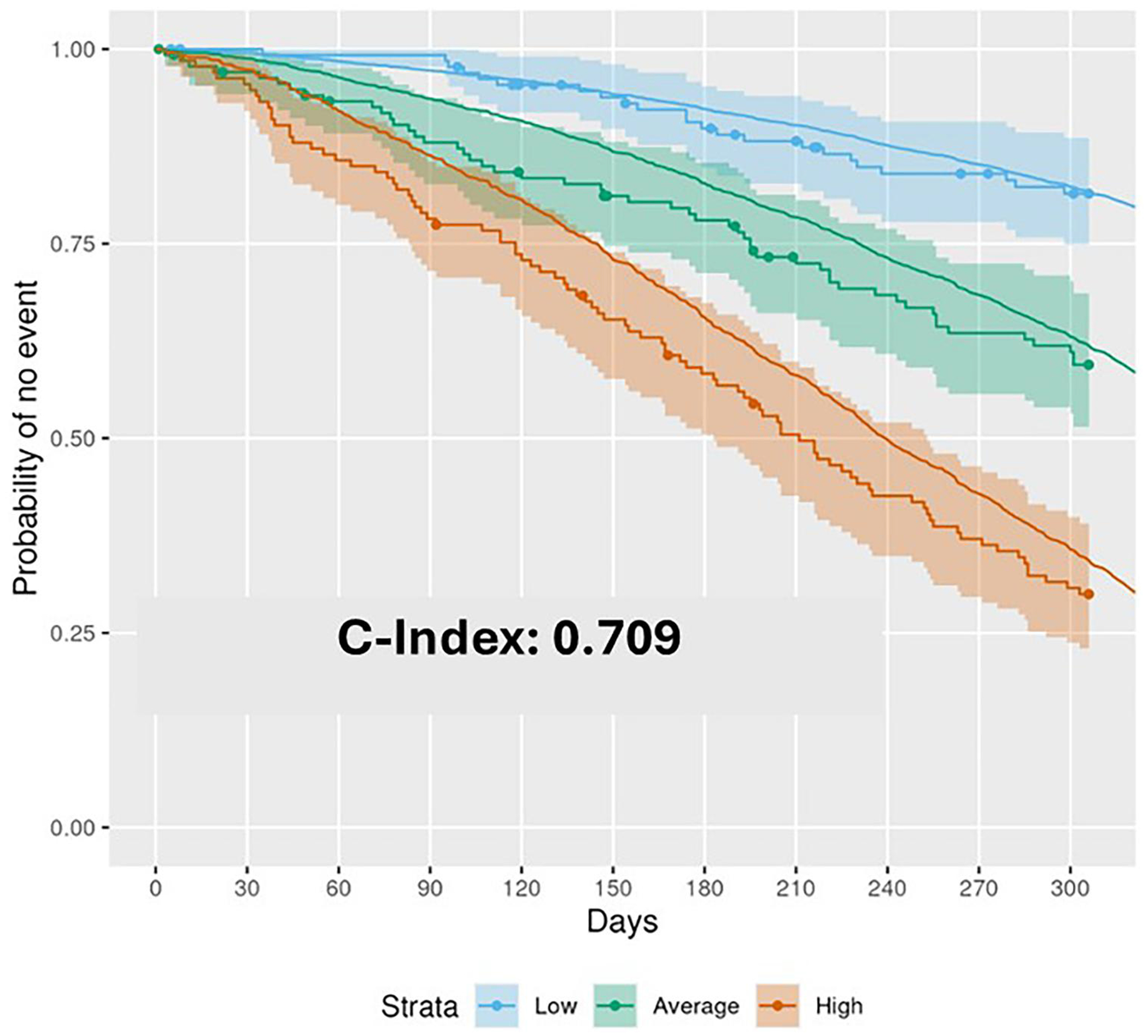
Kaplan-Meier (KM) curves for validation cohort tertiles. The smooth line for each tertile shows the median predicted value; the shaded colored area with the stepwise KM plots shows the 95% CI of the actual survival outcomes, which are shown by the corresponding dotted lines. The validation data was divided into tertiles, namely: low-risk (blue); average-risk (green); and high-risk (red). The C-index for the validation data is shown.

Figure 3 shows the GBM survival prediction model performance in terms of discrimination (C-index) and calibration (slope, CITL). The model’s ability to discriminate was strong and consistent across the 10 internal validation folds (shown in panel A), with an overall C-index of 0.821 (95% confidence interval [CI]: 0.811, 0.832). The model was well-calibrated on average, with a mean overall calibration-in-the-large value of −0.024 (−0.081, 0.033); however, the overall mean calibration slope was 1.101 (1.052, 1.149), thereby indicating an under-prediction of risk in high-risk patients and an over-prediction of risk in low-risk patients. As shown in Figure 3(B), testing of the external validation cohort yielded weaker yet still satisfactory discrimination with a C-index value of 0.709 (0.671, 0.746). There was a slight bias toward under-predicting risk at the external validation stage, as the calibration-in-the-large value was 0.083 (−0.073, 0.232); on the other hand, the calibration slope on external validation was 0.992 (0.789, 1.198), which was superior to the calibration slope on internal validation. The AUC at 306 days from baseline was 0.832 on internal validation, with high consistency observed between the 10-folds (Figure 4). Performance on the external validation cohort was slightly worse but still satisfactory (Figure 4) with an AUC of 0.757. Calibration plots demonstrated a consistent relationship between mean predicted survival probability and observed survival at Day 306 for both internal and external validation cohorts (see data in Supplementary Appendix 2). Finally, Figure 5 shows that survival predictions for each advancedstage tertile fell within the 95% CI of the observed KM curve, albeit showing a bias toward over-predicting survival in the average- and high- risk tertiles. Taken together, these results demonstrate that the model can predict survival probabilities with reasonable accuracy in advanced-stage ALS patients with severely impaired respiratory function.

## Discussion

The ability to predict outcomes in people living with ALS is critical, particularly in patients with advanced-stage disease, where such information is relatively deficient. Without more complete information, our collective clinical trial efforts to bring desperately needed novel therapeutics to pwALS will continue to be significantly constrained due in large part to disease outcome heterogeneity (32). In this research, we have extended previous efforts using various models to prognosticate ALS survival outcome (10,16,17,33). The relatively robust machine-learning model that we developed uses a substantial number of advanced-stage ALS patients and was externally validated on a dataset consisting exclusively of advanced-stage patients. It therefore improves the prognostication process for advanced-stage ALS, which will facilitate interpretation of single-arm phase 2 trials and enhance potential efforts to reduce cohort heterogeneity in randomized controlled trials.

The PRO-ACT dataset that we incorporated (*n* = 6896) was more representative of early-stage ALS (mean values for ALSFRS-R and VC% of 37.7 and 87.7, respectively). Therefore, we realized the importance of supplementing the training data with advanced-stage patients, which we accomplished by adding advanced-stage patients from the Emory University ALS Center (*n* = 678; mean ALSFRS-R and VC%, 23.5 and 37.2, respectively). To our knowledge, this is the first machine-learning model that incorporates extensive data from patients with advanced-stage ALS. Future efforts will be directed toward including additional advanced-stage ALS patients in the training set; specifically, it will be important to evaluate whether supplementing the PRO-ACT database with additional advanced-stage patients will augment the model’s sensitivity, specificity, and precision for survival prognostication in advanced-stage patients. The current reliance on predominantly early-stage patients may have contributed to the observed over-prediction of survival in the advanced-stage external validation cohort; furthermore, it is possible that a model trained exclusively on advanced-stage ALS patients might better predict survival outcomes in advanced-stage patients. However, given the current scarcity of advanced-stage data, such a model might have a limited performance and generalizability. Thus, our inclusion of early-stage patients from PRO-ACT greatly increased the size of the training set and improved generalizability to a wide spectrum of ALS patients.

Our selection process for enrichment, which was predicated upon obtaining data from individuals with at least one VC measurement of ≤50% predicted normal, indeed resulted in a patient population with disease that can be characterized as advanced based on other characteristics, namely, ALSFRS-R scores. However, going forward, it will be important to diversify the <50% VC training dataset with clinical parameters that were not represented in the current database, including: incorporation of individuals using mechanical ventilation (34,35), noninvasive ventilation (36–38), gastrostomy (39), and cough assist devices (40). Still yet, it is envisioned that future AI-driven models of survival prediction in advanced-stage ALS might optimally include biologic correlates such as markers of neurodegeneration (41) or neuroinflammation (4).

In the setting of advanced-stage ALS as we have operationally-defined the population here, survival remains the critical prognostication endpoint given the limitations in ALSFRS-R scores and vital capacity as clinical trial endpoints (23). Although predicting survival has some importance in clinical trials involving patients with early-stage ALS (20), it is critical for trials involving advanced-stage ALS patients and ultimately may be required for regulatory approval of candidate interventions. The machine learning survival prognostication model that we developed delivers in this regard, demonstrating good performance in the external validation cohort of advanced-stage ALS patients with a satisfactory C-index of −0.709 (0.671, 0.746), a good calibration slope of 0.992 (0.789, 1.198), and a slight bias to under-predict risk with a CITL of 0.083 (−.073, 0.232). The C-index value of 0.709 at the external validation stage was not as robust as the internal validation value; however, overall, the model performs adequately when generalizing outside of the training cohort. Given that, to our knowledge, no other prediction models have leveraged a substantial amount of advanced-stage ALS data, the model is particularly useful for prognostication in that population.

It should be noted that the ENCALS prognostication model by Westeneng et al. (15) reported stronger external validation with a C-index of 0.78 and a calibration slope of 1.01, which are more robust statistical values than the ones generated in our external validation efforts. We speculate that our reduced robustness in this regard may have been at least partially attributable to the substantial difference in patient population between the training and external validation datasets (e.g., the mean VC% in our two datasets were extremely divergent, at 83.2 *vs.* 39.2). In spite of this limitation, in our external validation cohort, which was comprised of only patients with ≤50% VC, our new model was able to stratify distinct tertiles of survival prognosis, including a good prognosis tertile that has a median projected survival probability of 85% at day 240 from baseline. Therefore, these data to some extent confirm the conclusions of prior investigations that highlighted enrichment for the slow-progressor phenotype in ALS patients who have advanced respiratory insufficiency (42). In sum, these data confirm that clinical trial consideration of patient heterogeneity is critical for both early- and advanced-stage ALS.

In future efforts, by using our model to select only patients in the lower two prognosticated-risk tertiles, we conclude that it is feasible to use survival as a primary endpoint in clinical trials evaluating patients with advanced-stage ALS. That is, the high-risk and average-risk tertiles have survival probabilities at day 240 post-baseline of 43 and 69%, respectively. With appropriate patient selection, candidate therapeutics could be reliably evaluated using a survival endpoint in just 8months with a single-arm phase 2 trial design using an AIdriven virtual control cohort. On the other hand, it is recognized that randomized controlled trials may also play an important role in the evaluation of novel interventions for the treatment of advanced-stage ALS. In such an RCT scenario, the survival prognostication model we developed can optimize clinical trial design by improving cohort homogeneity by risk stratification (13) and by including model predictions in the analysis of RCTs to reduce sample size or increase power (43).

Even though there are limitations to comparing across predictive models, it is important to further discuss the disease parameters that drive survival prognostication in advanced-stage ALS because such factors are quite different from the parameters that dictated disease outcome in the previous model trained exclusively on early-stage ALS patients (10). That is, in early-stage ALS, baseline ALSFRS-R scores (total and sub-scores) tended to have increased relative importance for determining outcome. By comparison, pulmonary parameters weighed heavily in the advanced-stage ALS prognostication, including baseline degree of VC deficiency and slope of decline in VC. Interestingly, age was the second most important factor driving the advanced-stage model (RI, 12.4%) but was only the 9th most important parameter in early-stage ALS patients (RI, 2.4%). Therefore, although the literature has described increasing age as an important risk factor for ALS (44), here we observed that this association may be particularly important in advanced-stage patients. Of interest, body mass index was a significant factor in the advanced-stage model, accounting for 4.2% relative importance of the survival predictions. Reduced BMI can result from a hyper-catabolic state, which has been described as an inflammatory component that drives ALS progression (45), perhaps especially in advanced-stage patients. Taken together, these results provide an initial understanding of the patient-specific factors that dictate clinical outcome in advanced-stage ALS that will assist in the design and interpretation of clinical trials in this setting.

It is important to discuss the potential limitations of our research findings. To avoid unreliable predictions caused by excessive imputation, we excluded subjects with more than five missing variables from the training set; while this exclusion was necessary, it potentially introduces bias into the training dataset if missingness is not random. Future research should evaluate model performance using alternative strategies for handling missing data. Another limitation of the current model is the potential for attrition bias in the VC_50_ datasets. That is, more severe patients may lack VC measurements below 50% due to death or inability to perform the test, thereby biasing the VC_50_ dataset toward slower progressors. Thus, while the current model was able to adequately predict survival outcomes in advanced-stage ALS, it may not accurately represent the trajectory of all advanced-stage ALS patients. The current model also did not incorporate the variable of respiratory failure (either mechanical ventilation or >22h per day of noninvasive ventilation usage); as such, the current model may have limited applicability in the subset of advanced-stage ALS patients with respiratory failure. However, given that the extent of ventilation usage is incorporated into the respiratory insufficiency subsection of the ALSFRS-R score, the effects of this limitation may be nominal. Finally, this model did not account for other known risk factors that would likely influence prognostication, including patient status for frontotemporal dementia or pathogenic C9orf72 repeat expansion, which were not consistently available in the training and validation data sources.

Patient heterogeneity has significantly constrained our collective efforts to bring new therapeutic modalities to the clinic for more effective treatment of ALS and remains an obstacle in both early- and late-stage patients. Here, we have successfully used a machine learning-based survival prognostication model to define the factors that drive such heterogeneity in advanced-stage ALS, thereby setting the stage for an increased ability to perform optimally-designed clinical trials in this unique patient population with a high rate of lethality that is universally excluded from current clinical trials.

## Supplementary Material

Supp 1

Supplemental data for this article is available online at https://doi.org/10.1080/21678421.2026.2652322

## Figures and Tables

**Table 1.** Demographics and baseline characteristics of ALS patient populations.

Characteristic^a^	PRO-ACT database (*n* = 6896)	Emory VC_50_ data (*n* = 678)	NHC VC_50_ data (*n* = 403)
Age			
Mean, *SD*	55.8 (11.5)	62.8 (12.0)	64.7 (11.3)
Q1, Median, Q3	48.0, 57.0, 64.0	55.1, 63.7, 71.8	59.0, 65.9, 71.6
Range	18–86	20–88	19–90
Sex (%; F/M)	37/63	45/55	43/57
Time from onset			
Mean, *SD*	655 (430)	986 (1121)	1163 (1124)
Q1, Median, Q3	364, 552, 831	406, 698, 1135	535, 814, 1413
Range	16-8741	69-14920	71-9771
Time from diagnosis			
Mean, *SD*	288 (322)	419 (656)	622 (875)
Q1, Median, Q3	91, 196, 377	0, 212, 552	132, 391, 729
Range	131-7372	365-7933	56-8335
Riluzole usage (%; Y/N)	76/24	87/13	55/45
Bulbar onset (%; Y/N)	22/78	30/70	37/63
Limb onset (%; Y/N)	76/24	64/36	57/43
ALSFRS-R			
Mean, *SD*	37.7 (5.3)	23.5 (8.0)	25.2 (7.7)
Q1, Median, Q3	34.0, 38.0, 42.0	18.0, 23.0, 29.0	19.5, 25.0, 31.0
Range	16–48	0–44	4–47
ALSFRS-R, slope of decline			
Mean, *SD*	0.6 (0.5)	1.2 (1.2)	0.9 (0.6)
Q1, Median, Q3	0.3, 0.5, 0.8	0.6, 0.9, 1.5	0.4, 0.7, 1.1
Range	0.0–5.4	0.1–14.2	0.1–4.7
%VC			
Mean, *SD*	87.7 (19.2)	37.2 (10.4)	39.2 (8.8)
Q1, Median, Q3	75.2, 87.5, 99.7	30.6, 39.9, 45.7	34.2, 41.5, 46.1
Range	10–291	3–50	8–50
Gastrostomy (%; Y/N)	2/98	24/76	28/72
Deaths through day 306	12% (851/6896)	50% (338/678)	41% (166/403)
Median survival (months)^b^			
From baseline	23.1 (22.6–24.3)	10.0 (8.6–12.1)	13.4 (11.9–15.7)
From diagnosis	44.3 (41.7–46.9)	23.0 (20.8–25.3)	30.6 (27.9–34.6)
From symptom onset	67.3 (63.6–71.3)	39.3 (36.6–42.5)	48.0 (43.3–53.3)

a Abbreviations: *SD*, standard deviation; F/M, female/male; Y/N, yes/no; % VC, vital capacity, percent of predicted normal; Q1, Q3, represent values at first and third quartile.

b Median survival, numbers in parentheses are lower and upper bound of the 95% confidence interval

## Data Availability

De-identified data generated for this study are available from the corresponding author on reasonable request, for up to three (3) years from publication or as required by the funder, subject to a data-use agreement. Requests for model use can be directed to the corresponding author or to Origent Data Sciences (frosado@origent.com ).
