# Supplementary figures and images for: Development of a machine learning-based survival prediction model for ALS inclusive of the advanced-stage population
Source: Amyotroph Lateral Scler Frontotemporal Degener. Author manuscript; Available in PMC 2026 May 5. (PMC13138003; doi:10.1080/21678421.2026.2652322)

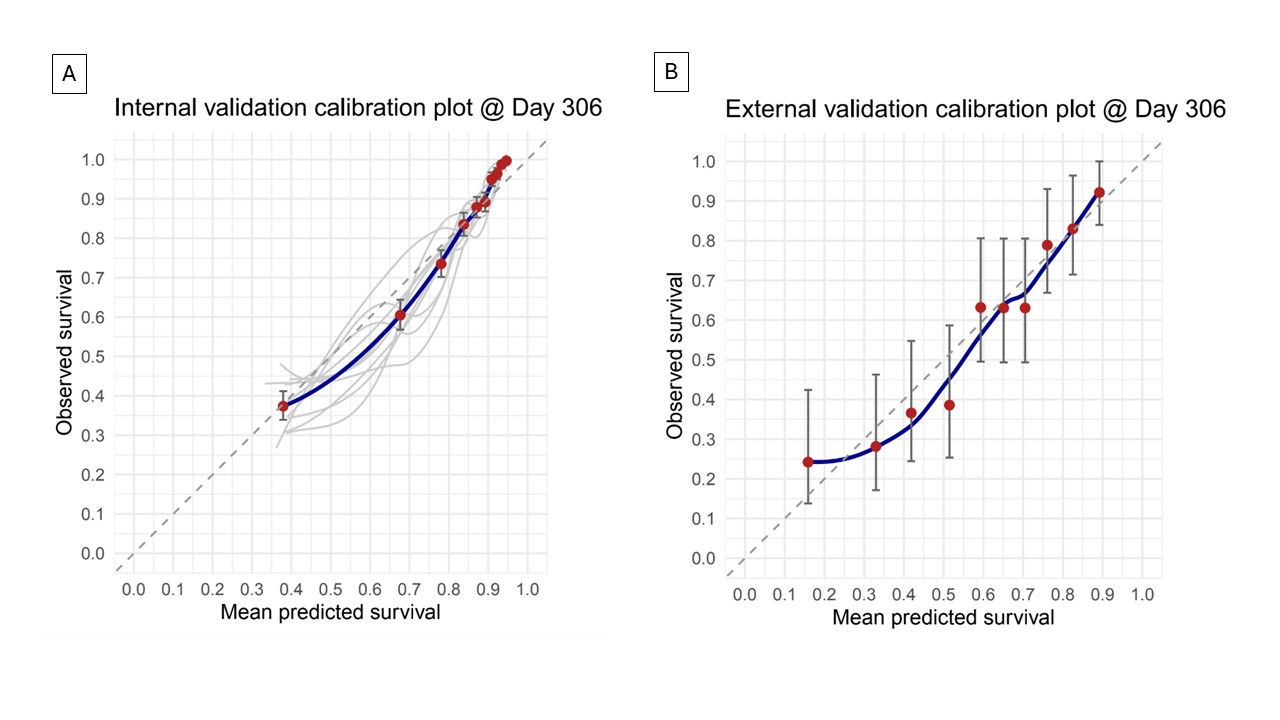

Supplement: Supp 1 [file NIHMS2165559-supplement-Supp_1.zip › Appendix 2.jpg]
